# OPA1 as a Cancer Target: Molecular Mechanisms, Structural Insights, and Strategies for Drug Development

**DOI:** 10.3390/antiox15010144

**Published:** 2026-01-22

**Authors:** Antonio Curcio, Ludovica Ganino, Ilenia Valentino, Massimo Gentile, Stefano Alcaro, Roberta Rocca, Anna Artese, Nicola Amodio

**Affiliations:** 1 Dipartimento di Scienze della Salute, Università Magna Græcia, 88100 Catanzaro, Italy; antonio.curcio@unicz.it (A.C.); alcaro@unicz.it (S.A.); artese@unicz.it (A.A.); 2 Dipartimento di Medicina Sperimentale e Clinica, Università Magna Græcia, 88100 Catanzaro, Italy; ludovicaganino@gmail.com (L.G.); ilenia.valentino30@gmail.com (I.V.); 3 Hematology Unit, Azienda Ospedaliera Annunziata, 87100 Cosenza, Italy; massimo.gentile@unical.it; 4 Department of Pharmacy, Health and Nutritional Science, University of Calabria, 87036 Rende, Italy; 5 Associazione CRISEA—Centro di Ricerca e Servizi Avanzati per l’Innovazione Rurale, Località Condoleo di Belcastro, 88055 Catanzaro, Italy

**Keywords:** OPA1, mitochondria, mitochondrial dynamics, mitochondrial dysfunction, SBVS, docking

## Abstract

Mitochondria are highly dynamic organelles that integrate metabolic regulation, signal transduction, and programmed cell death with their canonical role in adenosine triphosphate (ATP) production. Their ability to undergo continuous remodeling through the opposing processes of fusion and fission is essential for maintaining cellular homeostasis, preserving organelle quality control, and enabling adaptive responses to metabolic and oxidative stress. Among the core regulators of mitochondrial dynamics, the dynamin-related guanosine triphosphatase (GTPase) OPA1 plays a central role in inner membrane fusion, cristae architecture maintenance, bioenergetic efficiency, and the modulation of redox balance and apoptotic signaling. Accumulating evidence indicates that dysregulation of OPA1 expression or activity contributes to the initiation and progression of multiple malignancies, underscoring its importance in tumor cell survival, proliferation, metabolic adaptation, and resistance to stress. Here, we summarize current knowledge on OPA1 dysregulation in cancer and, based on preliminary, unpublished in silico analyses, we highlight the growing relevance of OPA1 as a therapeutic target, particularly through its GTPase domain and the still understudied Interface 7. Overall, these findings outline how integrated computational approaches could potentially guide the identification of novel OPA1 modulators, offering a conceptual framework that highlights OPA1 as a promising, yet still largely underexplored, target in oncology.

## 1. Mitochondrial Dynamics: An Overview

Mitochondria are highly dynamic and multifunctional organelles. Beyond their essential role in adenosine triphosphate (ATP) synthesis, they are integral to numerous cellular processes, including metabolic regulation, signal transduction, and the execution of programmed cell death [1]. A central aspect of mitochondrial function is their capacity to continuously alter shape, size, and intracellular distribution—a phenomenon referred to as mitochondrial dynamics. These dynamic processes enable mitochondria to rapidly adapt to the cell’s metabolic and physiological demands, thereby maintaining cellular homeostasis and facilitating responses to environmental or intracellular stress [2].

Mitochondrial dynamics are governed by two opposing yet complementary processes: fusion and fission. Mitochondrial fusion facilitates the mixing of contents between individual mitochondria, promoting the compensation of damage and the preservation of bioenergetic functionality [3]. This process is primarily regulated by mitochondrial guanosine triphosphatases (GTPases), including Mitofusins (Mfn1 and Mfn2), which mediate outer membrane fusion, and OPA1, which controls inner membrane fusion. In contrast, mitochondrial fission, orchestrated predominantly by the GTPase Dynamin-Related Protein 1 (DRP1) and its regulatory partners Mitochondrial Fission 1 protein (FIS1), Mitochondrial Fission Factor (MFF), and Mitochondrial Dynamics proteins (MiD49/51), enables the segregation of damaged mitochondria and their subsequent removal via mitophagy. Additionally, fission ensures proper organelle distribution during cell division, a process essential for cellular proliferation and differentiation [4].

The ability of mitochondria to remodel themselves through the mitochondrial dynamics machinery is crucial for multiple cellular processes, including the maintenance of pluripotency, the regulation of cell division and differentiation, the modulation of senescence, and the execution of programmed cell death [5]. Conversely, alterations in mitochondrial dynamics are associated with a huge variety of diseases, including neurodegenerative disorders, metabolic syndromes, cardiovascular diseases, and cancers, highlighting the critical role of these organelles in maintaining cellular and organismal health [6].

For this reason, targeting these nodes is considered promising; however, the heterogeneous nature of mitochondrial dynamics and the lack of a clear phenotypic threshold make it difficult to precisely define the mitochondrial contribution to different pathological contexts [7,8,9].

In this context, OPA1—a key mitochondrial dynamin-related GTPase—has emerged as a critical determinant of mitochondrial structural integrity, dynamics, bioenergetic capacity, and the regulation of redox and cell-death pathways. Dysregulation or aberrant expression of OPA1 has been increasingly linked to the pathogenesis of diverse malignancies [10,11,12].

Here, we summarize recent studies on the role of OPA1 dysregulation in tumorigenic processes. We highlight its expression patterns and functional roles in both solid and hematological malignancies, and critically discuss structural and mechanistic features that support OPA1 as a promising target for therapeutic intervention across diverse cancer types.

## 2. OPA1: Physiological Roles and Key Regulators

OPA1 is a GTPase of the mitochondrial inner membrane that plays a central role in controlling mitochondrial shape, cristae structure, and energetic function. It works in synergy with Mitofusins (MFN1/MFN2), which mediate outer membrane fusion, to regulate inner membrane fusion, oxidative phosphorylation (OXPHOS) efficiency, and the threshold for the release of pro-apoptotic signals as cytochrome C [13].

The OPA1 gene produces multiple isoforms through alternative splicing: the long forms (L-OPA1), anchored to the membrane, are essential for fusion and oligomerization, while the short forms (S-OPA1), derived from proteolytic cleavage, have modulatory roles that can either promote fusion or contribute to mitochondrial fragmentation depending on the context. The L-OPA1/S-OPA1 ratio is, therefore, critical for maintaining cristae integrity, respiratory capacity, and resistance to apoptosis [14].

OPA1 processing is regulated by mitochondrial metalloproteases such as OMA1 (activated under stress) and YME1L (active under homeostatic conditions), as well as indirect regulators like PARL, PHB/PHB2, and SLP2 [15]. The lipid composition of the inner membrane (IMM), particularly cardiolipin, also influences OPA1 activity and its oligomeric assembly necessary for fusion [16]. In mammalian cells, cardiolipin comprises approximately 18% IMM, whereas its abundance in the outer mitochondrial membrane (OMM) is less than 1% [17]. Certain isoforms are further involved in mtDNA regulation, contributing to repair and mitochondrial transcription/translation independently of fusion.

By continuously modulating the organization of the inner mitochondrial membrane and cristae architecture, OPA1 plays a crucial role in maintaining tissue homeostasis. This modulation optimizes respiratory efficiency and ATP production, limits basal oxidative stress, preserves mitochondrial genetic integrity, and establishes appropriate apoptotic thresholds [18]. Through this dynamic regulation of mitochondrial structure and bioenergetic function in response to physiological metabolic demands, OPA1 supports cellular and tissue homeostasis in energetically demanding organs such as muscle and nervous tissue. In this regard, OPA1’s role in maintaining the ultrastructure of cristae not only ensures the effective coupling of oxidative phosphorylation with ATP production but also stabilizes respiratory chain supercomplexes. This process helps to minimize electron leakage and decreases the local production of reactive oxygen species (ROS), thereby reducing basal oxidative stress and safeguarding tissues from metabolic harm [18].

Conversely, when OPA1 function is disrupted or imbalanced, it results in disorganized cristae, decreased respiratory efficiency, elevated oxidative stress, and impaired mitochondrial quality control. This situation ultimately makes cells and tissues more susceptible to metabolic dysfunction and increases their vulnerability to damage caused by stress, especially in organs that require high energy levels [19].

Alterations in OPA1 or its regulators lead to changes in mitochondrial morphology (hyperfusion or fragmentation), loss of energetic efficiency, mitochondrial DNA (mtDNA) dispersion, and increased cellular vulnerability. These phenomena are associated with neurodegenerative diseases, hereditary optic atrophy, metabolic dysfunctions, and tumorigenesis. Understanding these mechanisms is therefore crucial for interpreting cellular stress responses and identifying potential therapeutic targets [17]. In addition to the direct regulators of OPA1 mentioned above, p32/C1QBP acts as a crucial indirect modulator, preserving the functional form of OPA1 and preventing its excessive cleavage by OMA1. Predominantly localized in the mitochondrial matrix, p32 contributes to the cohesion and fusion of the mitochondrial network. Under physiological conditions, p32 maintains OPA1 in functional forms that ensure the integrity, flexibility, and respiratory activity of the mitochondrial network, whereas its deficiency activates OMA1 and generates incompetent short forms of OPA1 [16].

### Structural Features of OPA1

**Figure 1:**
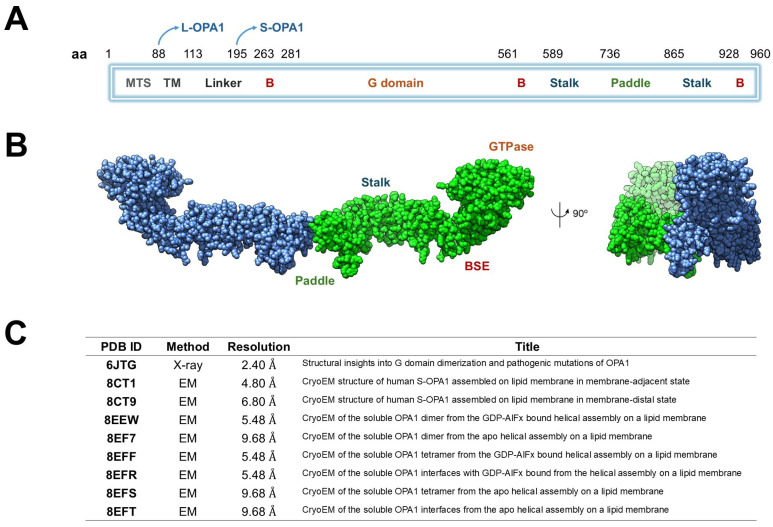
Structural organization and available structural data of human OPA1. (**A**) Schematic representation of OPA1 domain architecture. The protein includes a mitochondrial targeting sequence (MTS), transmembrane helix (TM), linker, B domains, GTPase domain, bundle signaling element (BSE), stalk, and paddle regions. The positions of the long (L-OPA1) and short (S-OPA1) isoforms are indicated. (**B**) Cryo-EM structure of the dimeric human OPA1 (chain A in blue and chain B in green) showing the main structural regions: the GTPase domain, the BSE, the stalk and paddle domains. A 90° rotated view is shown on the right. (**C**) Summary of available OPA1 structural entries in the Protein Data Bank (PDB), including experimental method, resolution, and title of each structure.

Structurally, OPA1 consists of several well-defined domains arranged sequentially along its polypeptide chain [11]. The N-terminal mitochondrial targeting sequence (MTS, ~residues 1–80) mediates the import of OPA1 into mitochondria, whereas the subsequent transmembrane (TM) segment (~residues 100–130) stably anchors the protein within the inner mitochondrial membrane (IMM). The central GTPase domain (~residues 200–600) mediates nucleotide binding and hydrolysis, providing the energy required for conformational changes during membrane remodeling. A middle or stalk domain (~residues 600–800) facilitates oligomerization and interdomain interactions, while the GTPase effector domain (GED, ~residues 800–960) modulates enzymatic activity and stabilizes higher-order assemblies. The C-terminal region further contributes to structural integrity and protein–protein interactions essential for OPA1 function (Figure 1A) [20].

L-OPA1 can be proteolytically cleaved by metalloproteases such as OMA1 (activated under stress) or YME1L (active under homeostatic conditions), generating a soluble short form (S-OPA1) that lacks the TM segment but retains the ability to oligomerize and promote membrane fusion (Table 1) [21]. L-OPA1 anchors to the IMM to initiate membrane tethering and fusion, whereas S-OPA1 is soluble in the intermembrane space and functions cooperatively with L-OPA1 to drive efficient membrane remodeling [11,22,23].

Biochemical and cryo-electron microscopy (cryo-EM) studies of S-OPA1 on cardiolipin-enriched membrane tubes (PDB IDs: 8EEW, 8EF7, 8EFS) reveal higher-order helical assemblies [22] (Figure 1B). In the 8EEW structure, S-OPA1 forms a helical lattice around lipid tubules in the presence of GDP-AlF_4_^−^, with three distinct interfaces: (i) between stalk domains of adjacent monomers, (ii) between the paddle region of one monomer and the stalk of its neighbor, and (iii) between membrane-inserting paddle helices of adjacent monomers. Comparison of apo (PDB ID: 8EF7) and nucleotide-bound (PDB ID: 8EEW) states shows subtle conformational shifts in the stalk–paddle interface, consistent with nucleotide-driven propagation of structural change from the GTPase domain outward toward the membrane-binding region [8EFS]. Collectively, these models (Figure 1C), along with PDB ID 6JTG [24], provide a mechanistic framework for OPA1, illustrating how GTPase dimerization communicates nucleotide state through the helix-bundle extension and stalk, and how the paddle domain engages the membrane to promote oligomerization, lattice formation, and IMM fusion [25].

**Figure 2:**
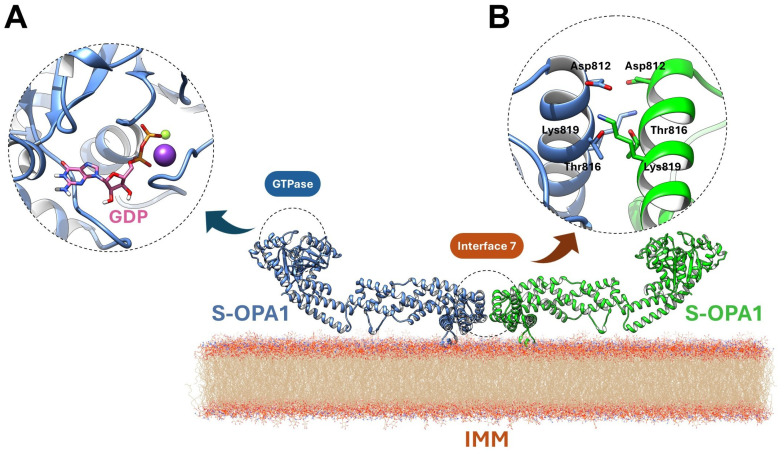
Structural features underlying OPA1 GTPase activity and Interface 7-mediated dimerization. (**A**) The nucleotide-binding pocket coordinates GTP (or GDP/BeF_3_^−^, pink) with Mg^2+^ (violet ball) and K^+^ (yellow ball), and is stabilized by conserved residues and catalytic waters, enabling specialized GTPase conformational dynamics. (**B**) Dimeric Interface 7 is formed by opposing paddle domains on cardiolipin-enriched membranes, with α-helices and loops mediating electrostatic contacts and salt bridges, linking GTP hydrolysis to conformational changes that drive IMM remodeling and fusion.

OPA1’s intrinsic GTPase activity is mediated by a conserved nucleotide-binding pocket accommodating GTP and coordinating Mg^2+^ and an unusual K^+^ ion [13]. GDP and the transition-state mimic BeF_3_^−^ are tightly ordered within the site [24]. Mg^2+^ is chelated by T302 (G1/P-loop), T323 (G2/Switch 1), and D398 (G3/Switch 2), while K^+^ is coordinated by S298, G317, E320, and the phosphate groups. A catalytic water adjacent to BeF_3_^−^, along with a bridging water linking Q297 and G401, completes the hydrolytic network (Figure 2A). Unique sequence features, including stabilization of the guanine base by T503 (β6–α5 loop), R316 (Switch 1), D470, and K468, and reinforcement of the extended Switch 1 segment by E320, R324, M321, and M322, confer specialized conformational dynamics distinct from other dynamin family members [26].

Although S-OPA1 lacks the TM segment, it retains partial IMM association via its paddle domain (residues I735–D869). The α1p and α4p helices and the L1p loop interact closely with cardiolipin-enriched bilayers, with K738, R857, and R858 mediating electrostatic contacts. High-resolution structural analyses further identify a dimeric Interface 7, formed by α3p helices of opposing paddle domains, stabilized by D812–K819 salt bridges and reinforced by T816 (Figure 2B) [24]. These features couple GTP hydrolysis to conformational rearrangements within the stalk and paddle domains, linking enzymatic activity to membrane deformation and establishing the molecular basis for OPA1-driven IMM fusion. Mutations in the GTPase, stalk, or paddle domains disrupt these processes, causing mitochondrial fragmentation and disease phenotypes such as dominant optic atrophy (DOA).

## 3. OPA1 in Cancer

The role of mitochondria in tumorigenesis is complex, involving energy metabolism, regulation of cell death, ROS production, autophagy processes, and drug responses [27].

Tumor cells actively manipulate mitochondrial dynamics—that is, the processes of fusion and fission—to meet their metabolic demands and promote proliferation and survival. Under physiological conditions, these mechanisms ensure energy homeostasis, redox balance, protein quality control, and sensitivity to apoptotic stimuli [17].

In tumors, this balance is often disrupted: increased fission has been reported in various carcinomas and is associated with more aggressive and proliferative phenotypes, whereas predominant fusion promotes mitochondrial stability and contributes to chemotherapy resistance [28]. This plasticity allows cancer cells to exploit both conditions to adapt and survive. Therefore, there is considerable interest in selectively modulating mitochondrial dynamics as a therapeutic strategy: inhibition of fission or promotion of fusion can induce apoptosis in fragmented tumors, while blocking fusion can sensitize resistant cells to treatment. The goal is not to suppress one process at the expense of the other, but to restore a physiological dynamic balance capable of limiting tumor plasticity while preserving the functionality of healthy tissues [28].

In this regard, the OMA1-OPA1 axis is emerging as a potential therapeutic target, as it is closely involved in the regulation of mitochondrial processes, including cell metabolism and apoptosis. Moreover, growing interest in OPA1 stems from its ability, beyond modulating cellular bioenergetics and apoptotic sensitivity, to regulate angiogenic and lymphangiogenic processes through ATP-independent mechanisms, likely linked to calcium signaling and the control of transcription factors such as NF-κB [29].

Cancer cells are characterized by a disrupted redox balance, marked by elevated levels of ROS alongside enhanced antioxidant defenses. This configuration enables tumor cells to exploit ROS-dependent signaling pathways while avoiding cell death caused by oxidative damage. Compared with normal cells, cancer cells experience increased ROS stress, partly due to oncogene activation and elevated metabolic activity [30]. Importantly, proteins involved in mitochondrial fusion have been associated with limiting excessive mitochondrial ROS production, thereby contributing to redox balance and promoting cell survival.

In this regard, OPA1 regulates mitochondrial ROS accumulation by maintaining cristae architecture and interacting with ATP synthase oligomers, thus playing a key role in mitochondrial redox homeostasis during respiratory activity [31]. From a functional perspective, OPA1’s role in shaping the tumor redox environment suggests that its increased expression may support cancer cell survival by enhancing the management of oxidative stress, in coordination with classical antioxidant systems such as glutathione and superoxide dismutase. This interplay between mitochondrial dynamics and redox adaptation may thus contribute to tumor metabolic plasticity and resistance to chemotherapy [31,32].

OPA1 expression has been evaluated in silico using large publicly available RNA-seq and proteomics datasets to assess its genetic alterations and correlation with patient prognosis, examining data from 33 tumor types [33]. Such multi-omics analyses revealed that OPA1 is overexpressed in several tumor types, including esophagus, lung, pancreas, and ovary. In some cases, such as clear cell renal carcinoma and ovarian adenocarcinoma, expression increases with tumor stage progression.

From a prognostic perspective, elevated OPA1 expression is frequently associated with poorer overall survival, as reported in breast, lung, pancreatic, and thyroid cancers. Conversely, in specific contexts, such as hepatocellular carcinoma, high OPA1 levels may correlate with more favorable outcomes [33]. A summary of current knowledge regarding OPA1 expression and its functional roles across cancer types is provided in Table 2 and discussed in the following sections.

### 3.1. OPA1 in Solid Tumors

In recent years, mitochondrial homeostasis has emerged as a key factor in the progression and treatment resistance of solid tumors. This dynamic balance within mitochondria is essential for maintaining cellular energy production, regulating apoptosis, and adapting to metabolic stress [34]. In this regard, OPA1 has gained increasing attention as a critical regulator of survival and progression in breast cancer, particularly due to its multifaceted roles in mitochondrial dynamics and function.

In triple-negative breast cancer (TNBC), which is known for its aggressiveness and resistance to conventional therapies, OPA1 is frequently overexpressed and associated with poor prognosis, suggesting that its modulation could represent an innovative therapeutic strategy [34,35]. Through integrated multidisciplinary approaches encompassing bioinformatic analyses, in vitro experimentation, and murine models, it has been demonstrated that OPA1 inhibition markedly attenuates tumor cell proliferation, migration, and invasiveness without impairing mitochondrial respiratory capacity. These findings indicate that the antitumor effects associated with OPA1 suppression arise from mechanisms more complex than a mere blockade of bioenergetic function [34,35].

Additional mechanistic insights derive from studies examining the adaptive responses that enable TNBC cells to withstand chemotherapeutic insult. Following DNA-damaging treatments, residual tumor cells exhibit pronounced mitochondrial elongation and enhanced OXPHOS, supporting the notion that mitochondrial remodeling constitutes a survival-promoting adaptation; conversely, taxane-based therapies diminish these mitochondrial features [36]. Notably, OPA1 inhibition counteracts this elongation and OXPHOS upregulation, thereby reducing the regenerative potential of residual cells and reinforcing the concept that OPA1-driven mitochondrial fusion represents a critical metabolic axis for post-chemotherapy persistence and survival [36].

Recent investigations in metastatic breast cancer further substantiate the pivotal role of OPA1 in facilitating tumor cell metabolic adaptation. In metastatic cells, OPA1 expression remains elevated despite reductions in proteins such as MFN1 and an overall increase in mitochondrial fragmentation, thereby sustaining the levels of OXPHOS required for enhanced proliferative and migratory capacity. Inhibition of OPA1, either through genetic ablation or selective pharmacological inhibitors, selectively impairs mitochondrial function in metastatic cells, diminishing their viability and metastatic dissemination while sparing non-metastatic and normal cells. This pronounced dependency on OPA1 underscores a critical therapeutic vulnerability, supporting the development of pharmacological strategies aimed at disrupting OPA1-mediated mitochondrial metabolism in metastatic disease, as further discussed in the following section [37].

Evidence from hepatocellular carcinoma (HCC) and cholangiocarcinoma (CCA) further highlights a consistent trend across diverse tumor types: mitochondrial fusion, predominantly mediated by OPA1 and MFN1, constitutes a critical node for tumor cell survival and proliferation [38]. Li et al. demonstrated that inhibition of OPA1 or MFN1 in liver tumors significantly reduces cell growth and tumorigenicity by inducing apoptosis and impairing mitochondrial bioenergetics. Analyses of tumor specimens and patient-derived organoids revealed pronounced mitochondrial hyperfusion relative to normal tissues, and gene silencing approaches targeting OPA1 or MFN1 disrupted this fusion, markedly decreasing both in vitro proliferation and in vivo tumorigenic potential. Notably, genetic or functional disruption of OPA1- and MFN1-mediated mitochondrial fusion resulted in elevated mitochondrial ROS, impaired metabolic efficiency, and reduced tumor growth, demonstrating that preservation of mitochondrial architecture is required to restrain pathological ROS accumulation and support tumor metabolism [34].

Metabolic profiling indicated that blockade of mitochondrial fusion primarily perturbs intracellular metabolic pathways, as corroborated by experiments showing reduced oxygen consumption and ATP production in tumor cells upon inhibition of mitochondrial fusion [38].

In non-small cell lung cancer (NSCLC), OPA1 is integral to maintaining resistance to Epidermal Growth Factor Receptor (EGFR)-targeted therapies. Cells exhibiting resistance demonstrate increased OPA1 expression, which sustains a hyperfused mitochondrial network and preserves narrow cristae architecture [39]. This configuration supports enhanced mitochondrial oxidative metabolism and reduces apoptotic susceptibility. Disruption of OPA1 function restores normal mitochondrial dynamics, facilitates cytochrome-c release, and re-sensitizes resistant cells to apoptosis induced by EGFR inhibition. Moreover, in EGFR inhibitor–resistant NSCLC models, high OPA1 levels and enhanced mitochondrial oxidative metabolism enable the cells to persist despite the treatment. Inhibiting OPA1 through pharmacological or genetic approaches can reinstate drug efficacy by modifying mitochondrial structure and energy production, reinforcing the notion that OPA1 aids resistant tumor cells in withstanding metabolic and oxidative stress [39]. These findings underscore OPA1 as a crucial regulator of mitochondrial remodeling and metabolic adaptation, which underlies therapeutic resistance in this tumor type [39].

Interactions between mitochondrial membranes—both inner and outer—and between mitochondria and other cellular organelles also play a critical role in determining ovarian cancer cell response to cisplatin and other chemotherapeutic agents. While the precise molecular mechanisms were not fully elucidated, recent evidence implicates the mitochondrial protease OMA1 in tumor progression [40].

In this context, the role of OMA1 in modulating chemotherapeutic response was investigated using ovarian cancer cell lines and subcutaneous tumor mouse models. Activation of OMA1 was found to enhance tumor cell sensitivity to cisplatin both in vitro and in vivo. Mechanistically, OMA1 mediates proteolytic cleavage of OPA1, leading to remodeling of mitochondrial cristae and alterations in inner membrane architecture. Concurrently, OMA1 cleaves DELE1, which translocates to the cytoplasm and engages the eukaryotic translation initiation factor 2-alpha kinase 1 (EIF2AK1). This interaction cooperates with the endoplasmic reticulum (ER) stress sensor eukaryotic translation initiation factor 2-alpha kinase 3 (EIF2AK3), amplifying downstream signaling through EIF2S1 phosphorylation and Activating Transcription Factor 4 (ATF4) activation. These events collectively contribute to outer mitochondrial membrane destabilization and the induction of apoptosis.

The work by Herkenne et al. highlighted an additional role for OPA1 in tumor angiogenesis, offering new insights into how mitochondrial function contributes to cancer progression through promoting neovascularization, which in turn supports both tumor growth and metastatic spread [41].

In the context of endothelial cells, OPA1 maintains an optimal metabolic profile, ensuring sufficient ATP production and tight regulation of oxidative stress. In addition, OPA1 indirectly modulates the transcription of pro-angiogenic genes through the NF-κB signaling pathway: its absence triggers aberrant activation of this pathway, disrupting proper gene expression and impairing angiogenic capacity. This interplay between mitochondrial function and nuclear signaling underscores the dual role of OPA1 in regulating both endothelial metabolism and the plasticity of endothelial cells within the tumor microenvironment [41,42].

### 3.2. OPA1 in Hematologic Malignancies

Hematologic malignancies exhibit a pronounced dependence on mitochondrial structure and bioenergetic function. Numerous studies indicate that disorders such as multiple myeloma [43,44,45], leukemias and lymphomas [46,47], dysregulate the mitochondrial dynamics machinery for prompting metabolic rewiring, tumor growth and drug resistance. Leukemias, in particular, rely on mitochondrial integrity, characterized by high OXPHOS, organized cristae architecture, and tight regulation of apoptotic thresholds. Within this context, OPA1 has emerged as a critical mitochondrial factor, as leukemic cells require well-structured cristae, sustained OXPHOS, and controlled apoptotic signaling to support proliferation and resist cytotoxic therapies [47].

Acute myeloid leukemia (AML) blasts and leukemic stem cells (LSCs) are heavily reliant on OXPHOS-driven proliferation, a metabolic state dependent on intact cristae and efficient assembly of electron transport chain (ETC) supercomplexes. Several studies have underscored the pivotal role of OPA1 in maintaining this structural and functional integrity. Chen et al. demonstrated that disruption of cristae architecture through deletion of CLPB, an AAA^+^ ATPase that interacts functionally with OPA1, induces mitochondrial stress, cytochrome c release, and sensitization to the BCL-2 inhibitor Venetoclax. This work highlighted that AML cells maintain Venetoclax resistance not only through anti-apoptotic proteins but also via preservation of OPA1-dependent cristae [42].

Further supporting this model, Larrue et al. showed that inhibition of mitochondrial fusion, achieved through genetic suppression of OPA1 or MFN proteins, causes mitochondrial dysfunction, reduces OXPHOS, and impairs leukemic cell viability both in vitro and in vivo [48]. In patient-derived xenograft models, MFN2 loss impaired AML cell reconstitution and promoted differentiation, underscoring that mitochondrial fusion supports not only metabolism but also stemness maintenance in leukemia [48]. Consistently, the prototypical OPA1 inhibitor MYLS22 induced cell cycle arrest in AML by promoting mitochondrial dysfunction–driven ROS accumulation, which activates cellular stress responses and blocks cell cycle progression at the G1/S transition [48]. Complementing these findings, La Vecchia et al. identified selective small-molecule OPA1 inhibitors that remodel cristae architecture, decrease respiratory competence, and re-sensitize drug-resistant AML cells to cytotoxic therapies [49].

Mechanistic integration with oncogenic signaling was further demonstrated by Sharma et al., who showed that ERK1/2 inhibition induces BAX-dependent OPA1 proteolysis, leading to cristae widening, mitochondrial depolarization, and enhanced Venetoclax sensitivity [50]. These results suggest that AML resistance can be overcome not only by perturbing the ETC but also through BAX-dependent proteolytic processing of OPA1 [48]. Collectively, these studies establish the OPA1–caseinolytic peptidase B (CLPB) cristae-maintenance axis as a central survival pathway in AML and a promising therapeutic target also in the drug-resistant setting.

In contrast, T-cell acute lymphoblastic leukemia (T-ALL) exhibits a distinct mitochondrial phenotype, driven by high metabolic demand and vulnerability to oxidative stress. In pediatric T-ALL, approximately 20% of cases display glucocorticoid resistance, accompanied by increased mitochondrial mass, elevated OXPHOS capacity, and reliance on intact fusion machinery to maintain redox homeostasis. OPA1 is central to this stress-buffering system [51,52]. Pharmacological co-modulation of mitochondrial function using NS1619, a BK-K^+^ channel activator, and DHEA, a pentose phosphate pathway inhibitor, significantly increased mitochondrial ROS, selectively inducing apoptosis in T-ALL cells while sparing normal thymocytes. Mechanistically, mitochondrial ROS activated OMA1, leading to OPA1 cleavage, disruption of mitochondrial fusion, and extensive cristae remodeling [53]. In this context, OPA1 functions as a crucial downstream effector of oxidative stress, wherein its ROS-dependent inactivation transforms mitochondrial redox imbalance into structural collapse and apoptotic cell death. Consequently, ROS-induced OPA1 cleavage constitutes a pivotal mechanistic step that connects oxidative stress to selective mitochondrial dysfunction and tumor cell elimination in T-ALL [53].

These findings indicate that, unlike in AML, T-ALL cells depend on OPA1 primarily to mitigate ROS, preserve cristae integrity, and prevent mitochondrial fragmentation under stress. Transcriptomic analyses further reinforce the importance of mitochondrial dynamics in leukemic progression: high expression of the fission regulator MFF correlates with poor prognosis in AML, suggesting that imbalances in fusion–fission dynamics contribute to disease aggressiveness [54].

In diffuse large B-cell lymphoma (DLBCL), there is a notable dependence on mitochondrial quality control pathways, as these tumors require intact oxidative phosphorylation and stress-adaptive signaling to maintain their rapid proliferation. Within this context, OPA1 plays a crucial role in mitochondrial homeostasis by preserving cristae structure and fusion dynamics, thereby facilitating efficient respiratory function and mitigating metabolic stress [55]. Consistently, OMA1-dependent proteolytic cleavage of OPA1 impairs mitochondrial quality control signaling and disrupts the ATF4-mediated Integrated Stress Response, ultimately reducing the capacity of DLBCL cells to adapt to nutrient and proteotoxic stress [55].

Altogether, these studies establish OPA1 as a shared mitochondrial vulnerability across hematologic malignancies, highlighting the therapeutic potential of approaches that destabilize cristae architecture, modulate the OMA1–OPA1 pathway, or combine mitochondrial remodeling with BCL-2 inhibition.

## 4. Therapeutic Strategies for OPA1 Inhibition in Cancer

Strategies to modulate OPA1 function include small-molecule inhibitors, such as MYLS22, a small-molecule selective inhibitor of OPA1. It was identified through a high-throughput screening of a library of approximately 10,000 small molecules, and it exhibits reversible and non-competitive activity against OPA1 GTPase function [12].

**Figure 3:**
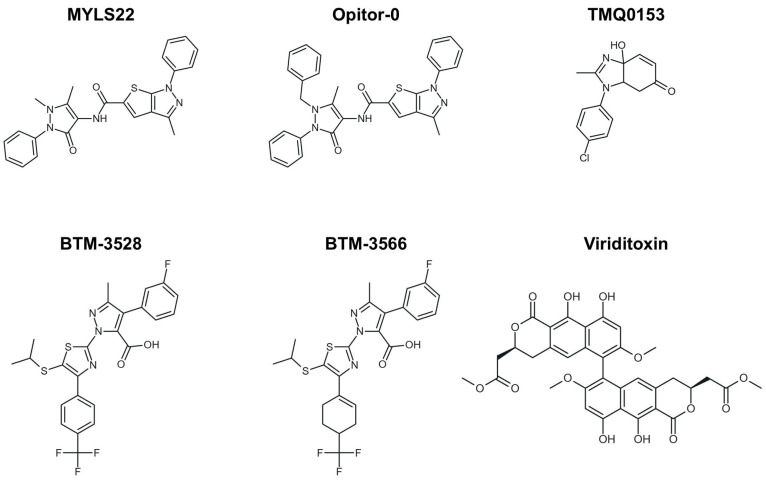
Small-molecule modulation of OPA1 and mitochondrial dynamics. Two-dimensional structures of the representative OPA1 inhibitors, including MYLS22, Opitor-0, TMQ0153, BTM-compounds (BTM-3528 and BTM-3566), and viriditoxin (VDT), are shown.

From a chemical perspective, MYLS22 is a heterocyclic derivative (thieno-pyrazole with an antipyrine group) and is listed in chemical databases under CAS 306959-01-3 (Figure 3). It has subsequently served as a prototypical compound in structure-activity relationship (SAR) studies, guiding the development of optimized inhibitors such as Opitor-0 (Figure 3) [12].

Techniques such as Saturation Transfer Difference NMR (STD-NMR) confirmed direct interactions between MYLS22 and the GTPase domain of OPA1, while molecular docking placed MYLS22 within the GTP-binding pocket of OPA1, overlapping the GDP site, with the phenyl-thienopyrazole moiety occupying the guanine recognition region [39].

MYLS22 has been shown to induce mitochondrial cristae remodeling, promote cytochrome C release, and increase the sensitivity of tumor cells to pro-apoptotic therapies, without significant toxicity in non-tumor cells in preclinical models [39].

Subsequent optimization efforts led to the development of Opitor-0, a more potent and soluble derivative that further enhances cytochrome c release and sensitizes tumor cells to Bcl-2 inhibition. In triple-negative breast cancer, MYLS22 and Opitor-0 were non-cytotoxic alone but synergized strongly with ABT-737, with Opitor-0 showing four-fold greater activity [12].

This enhanced activity is consistent with the SAR data, which identify three key determinants of potent OPA1 inhibition: (i) the presence of a phenyl group on the thieno-pyrazole scaffold, (ii) a negatively charged substituent positioned to engage the OPA1 GTPase pocket, and (iii) specific modifications to the antipyrine moiety. Compounds lacking the phenyl substituent (Opitor-10 to Opitor-15) or missing the antipyrine group entirely (Opitor-5) were inactive, underscoring the essential role of both features. Conversely, replacing the antipyrine N-methyl with a benzyl group in Opitor-0 enhanced inhibitory activity (IC_50_ = 3.0 ± 1.6 μM). Opitor-3 demonstrated that a carboxylate can act as a phosphate bioisostere, achieving higher potency with reduced maximal inhibition [12].

Together, these findings not only validate the chemical features required for effective OPA1 inhibition but also reinforce the concept that OPA1 represents a promising therapeutic target to overcome resistance to anti-apoptotic drugs. Inhibiting OPA1 not only disrupts mitochondrial fusion but also directly regulates cytochrome c release, thereby amplifying responses to Bcl-2–based therapies [12].

In the studies reported by Noguchi et al., OPA1 inhibitors such as MYLS22 and Opitor-0 (Figure 3) were tested in models of NSCLC resistant to gefitinib, particularly the PC9M2 cell line, which harbors an EGFR mutation conferring resistance. OPA1 inhibition led to profound mitochondrial remodeling and fragmentation, increased cytochrome C release, and apoptosis in resistant cells. In mouse models, combining gefitinib with MYLS22 significantly reduced orthotopic tumor growth, suggesting that it can restore sensitivity to anti-EGFR therapies [39].

In AML, recent studies demonstrated that inhibiting mitochondrial fusion through either genetic approaches or the OPA1 inhibitor MYLS22 exerts profound anti-leukemic effects. Treated cells undergo structural mitochondrial remodeling, reduced OXPHOS, decreased ROS levels, G_0_/G_1_ cell cycle arrest, and loss of self-renewal capacity. Importantly, these changes do not significantly impair normal hematopoietic progenitors, highlighting the selectivity and therapeutic window of this strategy [48].

Similarly, Park et al. explored the tetrahydrobenzimidazole compound TMQ0153 (Figure 3) in AML. TMQ0153 reduced OPA1 and MFN2 levels, disrupted mitochondrial function, induced metabolic reprogramming, and triggered caspase-dependent apoptosis; in xenograft models, it reduced tumor burden and synergized with Venetoclax or azacitidine to prolong survival [56].

As stated before, aggressive DLBCLs also exhibit vulnerability to mitochondrial stress modulation. A novel class of compounds, known as BTM-compounds (BTM-3528 and BTM-3566) (Figure 3), activates the mitochondrial protease OMA1. This activation results in the cleavage of DELE1 and OPA1, mitochondrial fragmentation, Heme-Regulated Inhibitor (HRI) kinase activation, and α-subunit of eukaryotic initiation factor 2 (eIF2α) phosphorylation, ultimately leading to growth arrest and apoptosis. Low expression of the negative regulator FAM210B confers tumor selectivity, and oral BTM-3566 induced complete tumor regression in multiple xenograft models [54].

Finally, viriditoxin (VDT)-a fungal-derived polyketide natural product with known cytotoxic properties-exemplifies the therapeutic potential of directly targeting mitochondrial architecture (Figure 3). VDT disrupts mitochondrial respiration, induces membrane depolarization, and promotes cytochrome c release through OPA1 cleavage, highlighting mitochondrial dynamics controlled by OPA1, MFN2, and CLPB as therapeutic vulnerabilities in hematologic malignancies beyond metabolic targeting [56,57].

Given that OPA1 has only recently emerged as a pharmacological target, current knowledge of the off-target effects associated with OPA1-directed small molecules remains limited. A major challenge in the development of GTPase inhibitors is their potential lack of selectivity, stemming from the high degree of structural conservation within the GTPase domain, as observed among members of the dynamin superfamily [12]. Moreover, because OPA1 plays a central role in maintaining mitochondrial architecture and bioenergetic homeostasis, its broad or sustained inhibition could theoretically impair mitochondrial function in non-malignant tissues, particularly under conditions of metabolic demand or cellular stress [58,59]. Collectively, these considerations suggest that the current scarcity of data on off-target effects primarily reflects the early stage of OPA1-targeted drug discovery.

### Computational Insights and Emerging Perspectives on OPA1 Drug Targeting

**Figure 4:**
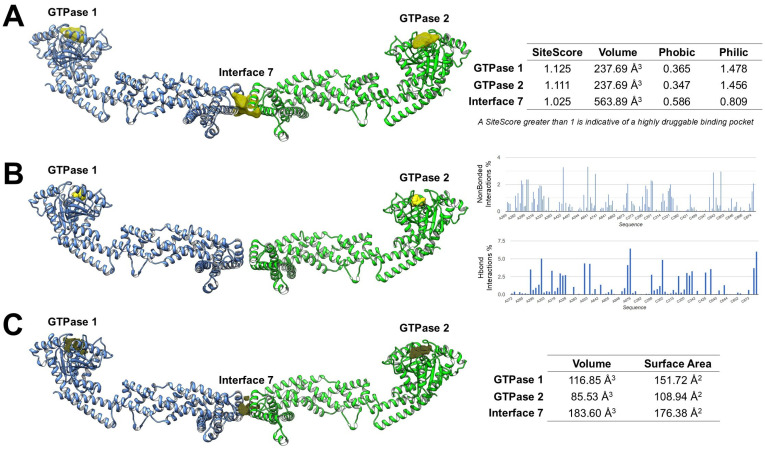
Computational analysis of OPA1 druggable pockets. (**A**) SiteMap reveals the two GTPase pockets as highly druggable polar sites, whereas Interface 7 is more hydrophobic and less polar. (**B**) FTMap shows high surface areas at the GTPase pockets, while Interface 7 is undetected, indicating a shallow or transient site. (**C**) KVFinder reveals pocket volumes (GTPase 1: 116.85 Å^3^, GTPase 2: 85.53 Å^3^, Interface 7: 183.60 Å^3^), suggesting Interface 7 could fit bulkier ligands.

Despite growing recognition of OPA1 as a central regulator of mitochondrial dynamics and its emerging implications in cancer biology, the development of small-molecule modulators targeting this protein remains at an early stage. To date, only a limited number of experimental ligands have been reported [12], highlighting a broader scarcity of validated inhibitors and emphasizing the value of computational strategies in supporting drug discovery efforts. In this context, in silico approaches, including binding site prediction, molecular docking, and druggability assessment, could provide a rational framework for identifying structural features amenable to ligand engagement and for prioritizing regions suitable for structure-based drug design [60,61,62]. Several computational tools, such as SiteMap [63], FTMap [64,65], and KVFinder [66], enable systematic identification and characterization of potential binding pockets based on geometry, polarity, and interaction potential. Using the high-resolution OPA1 structure (PDB 8CT9) [25], our integrated, unpublished analyses suggest that the two canonical GTPase nucleotide-binding pockets are predicted to be druggable by multiple computational tools. In contrast, Interface 7, corresponding to the α3p paddle–paddle interaction site in S-OPA1, was detected only by SiteMap and KVFinder, suggesting that it may represent a shallower or more transient interface less amenable to fragment-based recognition by FTMap (Figure 4).

SiteMap analysis predicted that both GTPase pockets exhibit SiteScores greater than 1, a threshold typically associated with highly druggable binding sites. Both GTPase 1 and GTPase 2 binding sites exhibited lower Phobic scores than Philic scores. These profiles hypothesize a predominantly polar binding environment, favoring the formation of hydrogen bonds and electrostatic interactions, features that support engagement by small-molecule ligands. In contrast, Interface 7 showed a higher Phobic score and a lower Philic score, reflecting a more hydrophobic environment than the GTPase sites. This suggests that ligands targeting Interface 7 may require stronger hydrophobic or amphipathic characteristics and could be less amenable to classical polar-driven binding compared to the nucleotide-binding pockets (Figure 4A).

FTMap mapping supported these predictions by visually identifying regions of high probe density at the GTPase sites, supporting their suitability for ligand engagement. Quantitative assessment of non-bonded interactions and hydrogen-bond interactions showed that the GTPase pockets could possess a high proportion of polar contacts (HBond interactions ~40–45%) alongside significant van der Waals contributions, consistent with favorable drug-binding environments. However, FTMap did not detect Interface 7 (Figure 4B).

KVFinder further characterized pocket geometry and volume, providing additional insights into their druggability. GTPase 1 has a volume of 116.85 Å^3^, GTPase 2 of 85.53 Å^3^, and Interface 7 of 183.60 Å^3^. The larger volume of Interface 7, despite its reduced polar score, suggests it could accommodate bulkier ligands or fragments designed to disrupt the paddle dimer interface (Figure 4C).

**Figure 5:**
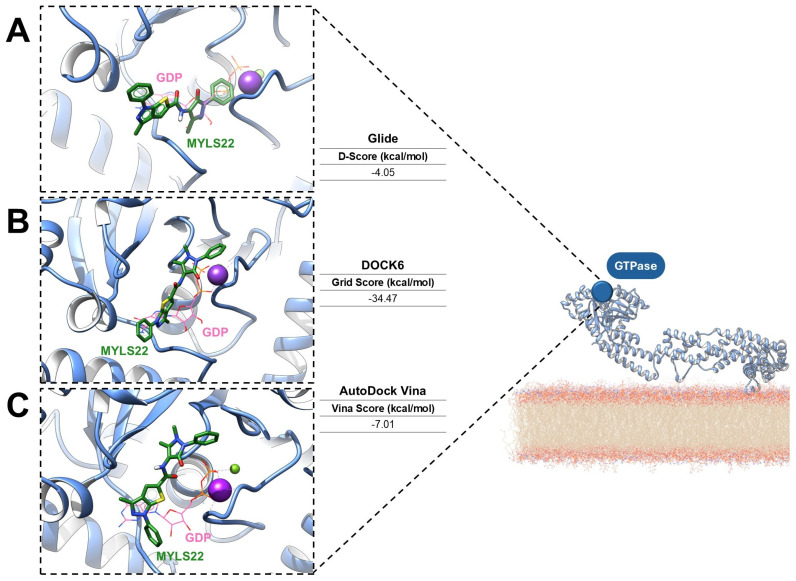
Predicted binding modes of MYLS22, a representative OPA1 GTPase inhibitor (green sticks), docked into the crystal structure of human OPA1 in its GDP-bound state (PDB ID: 6JTG; light-blue cartoon). The binding poses were generated using (**A**) Glide (Schrödinger), (**B**) DOCK6 (UCSF), and (**C**) AutoDock Vina. GDP is shown as orange sticks, while magnesium and potassium ions are represented as purple and green spheres, respectively.

To provide a perspective for assessing druggability in mitochondrial GTPases, preliminary molecular docking analyses of the OPA1 GTPase domains were carried out via three established computational platforms: Glide version 7.8 [67,68], DOCK6 version 6.13 [69,70], and AutoDock Vina version 6.13 [71]. This comparative application suggests how complementary docking algorithms can identify conserved binding interactions and guide the rational design of small-molecule modulators. The ligand MYLS22 was selected as an ideal inhibitor of the GTPase domain, and molecular docking was performed using the PDB structure 6JTG, chosen because of its high-resolution definition of the GTPase active site, the presence of the GTP cofactor, and coordination of the catalytic metal ions [24]. For all docking workflows, a grid centered on the GTP cofactor was generated. Across the different docking programs, MYLS22 consistently localized within the GTP-binding pocket, forming stabilizing interactions with conserved residues. Notably, in each case, superposition of MYLS22 with the co-crystallized GTP revealed that the phenyl-1H-thieno [2,3-c]pyrazole moiety of MYLS22 occupies a spatial position comparable to the ribose–base region of GTP (Figure 5), as reported in the literature [12].

Glide SP protocol yielded a docking score of −4.05 kcal/mol (Figure 5A), whereas DOCK6 reported grid-based interaction energies of −34.47 kcal/mol (Figure 5B). AutoDock Vina predicted a binding affinity of −7.01 kcal/mol (Figure 5C). Although the scoring metrics are not directly comparable, the binding orientations are consistent. The main variations concern the orientation of the 1,5-dimethyl-3-oxo-2-phenyl-2,3-dihydro-1H-pyrazol-4-yl substituent. All best docking poses consistently place the antipyrine moiety in an orientation that mimics the GTP triphosphate region. Glide, DOCK6, and AutoDock Vina align this group within the phosphate recognition subsite bordered by the G1/P-loop and G2/Switch 1, in agreement with previous literature [12]. In the DOCK6 pose, the oxo group additionally coordinates the catalytic potassium ion, further supporting a catalytically relevant interaction pattern. Despite minor tool-dependent differences, all docking algorithms predict favorable and coherent interactions within the OPA1 GTPase domain, reinforcing both its accessibility and high druggability. Collectively, these results support MYLS22 as a robust starting scaffold and highlight the OPA1 GTPase pockets as structurally well-defined targets for small-molecule inhibition.

In addition to the catalytic site, Interface 7 may represent a secondary allosteric region suitable for fragment-based or structure-guided design.

Collectively, this methodological convergence provides a promising framework for advancing OPA1-focused drug discovery in cancer and other mitochondrial dysfunction-related disorders.

## 5. Conclusions

Mitochondria are central hubs of cellular metabolism, stress adaptation, and apoptosis, and their continuous remodeling through fusion and fission is essential for maintaining organelle integrity and overall cellular homeostasis. Among the key regulators of these dynamic processes, OPA1 is a critical determinant of inner mitochondrial membrane fusion, cristae structure, bioenergetics, and redox control. Increasing evidence shows that aberrant OPA1 expression or activity contributes to the initiation, progression, and therapeutic resistance of numerous solid and hematologic malignancies. Despite its clear relevance in cancer biology and its association with poor clinical outcomes, OPA1 remains an underexplored therapeutic target [30,42].

At present, efforts to develop small-molecule modulators of OPA1 remain at a very early stage, with only a few experimental ligands reported [12,49,56]. This scarcity highlights the need for innovative strategies to accelerate target characterization and ligand discovery. In this context, computational approaches offer a powerful, cost-effective means to identify druggable pockets, guide rational inhibitor design, and prioritize therapeutic hypotheses.

Our preliminary in silico analyses support the druggability of OPA1 and highlight the GTPase domain as a particularly promising site for small-molecule modulation. Systematic mapping of putative pockets identified cavities with highly favorable structural and physicochemical properties. Consistently, docking studies positioned the known inhibitor MYLS22 within the conserved GTP-binding pocket, reproducing the canonical interaction pattern observed for native nucleotides. These observations are consistent with existing structural data and provide a solid foundation for leveraging this region in future drug-design efforts [12]. Moreover, a comparatively understudied region named Interface 7, originally described by von der Malsburg et al. [25] and implicated in OPA1 dimerization, emerged as a potentially druggable site deserving further investigation.

Together, these findings strengthen the rationale for pursuing OPA1 as a novel anticancer target and lay the groundwork for future screening campaigns. Expanding in silico prediction through large-scale docking, pharmacophore modeling, molecular dynamics simulations, and thermodynamic profiling will be crucial for identifying new chemical scaffolds capable of modulating OPA1 function. Finally, the integration of structural biology, computational chemistry, and experimental validation will be essential to advance OPA1-directed therapeutics from conceptual exploration toward tangible anticancer strategies.

## Figures and Tables

**Table 1:** Structural and functional distinctions between long (L-OPA1) and short (S-OPA1) forms of OPA1.

Feature	L-OPA1 (Long Form)	S-OPA1 (Short Form)
**Structure**	Contains the N-terminal TM domain anchoring it to the IMM.	Lacks the TM domain due to proteolytic cleavage at specific sites (S1 by OMA1, S2 by YME1L).
**Localization**	Membrane-bound, tightly associated with IMM.	Soluble in the intermembrane space; loosely associated with IMM.
**Function**	Essential for membrane tethering and fusion. Provides scaffold for oligomerization.	Facilitates fusion cooperatively with L-OPA1; may regulate cristae remodeling.
**Processing**	Generated first from the full-length precursor.	Produced by site-specific cleavage of L-OPA1 by mitochondrial proteases (OMA1/YME1L).
**Oligomerization**	Forms L-OPA1 homotypic or L-OPA1/S-OPA1 heterotypic oligomers necessary for inner membrane fusion.	Cannot anchor alone; functions mainly in combination with L-OPA1.

**Table 2:** Opa1 expression, functional role and therapeutic implication across cancer types.

Cancer Type	OPA1 Expression/Status	Functional Role in Tumor Biology	Therapeutic Implications
**Breast Cancer**	Frequently overexpressed; higher levels correlate with poor prognosis.	Supports mitochondrial fusion, cristae maintenance, OXPHOS capacity, migration, and invasion; promotes survival after chemotherapy.	OPA1 inhibition reduces proliferation, migration, and metastasis; restores sensitivity to apoptosis-inducing agents.
**Non-Small Cell lung cancer**	Often upregulated; associated with resistance to to Epidermal Growth Factor Receptor (EGFR) inhibitors.	Maintains mitochondrial integrity in resistant clones.	OPA1 inhibitors (e.g., MYLS22) restore gefitinib sensitivity and trigger apoptosis.
**Ovarian Cancer**	Altered OMA1-OPA1 axis; OMA1 activation leads to OPA1 cleavage.	OPA1 cleavage mediates cristae remodeling during chemotherapy response; participates in stress-induced apoptosis.	OMA1 activation increases cisplatin sensitivity via OPA1 processing.
**Hepatocellular carcinoma**	OPA1 overexpression observed contributes to tumor progression.	Supports mitochondrial fusion, OXPHOS metabolism, tumor growth.	Silencing OPA1 reduces proliferation, ATP production, and tumorigenicity.
**Cholangiocarcinoma**	Increased OPA1/MFN1 activity promoting mitochondrial fusion.	Enhances bioenergetic efficiency and supports tumor proliferation.	Targeting fusion machinery impairs tumor growth.
**Renal Cell Carcinoma**	OPA1 expression increases with tumor stage.	Potential role in metabolic rewiring and disease progression.	Prognostic biomarker candidate.
**T-cell Acute Lymphoblastic Leukemia**	OPA1 is cleaved during ROS-induced mitochondrial stress.	Loss of OPA1 function triggers mitochondrial fragmentation and apoptosis.	Modulating OMA1–OPA1 axis selectively kills leukemic cells via oxidative stress.
**Acute Myeloid Leukemia**	OPA1 contributes to therapy resistance (e.g., to Venetoclax).	Supports cristae structure, mitochondrial integrity, and survival.	Genetic targeting or pharmacological inhibition of OPA1 restores drug sensitivity; OPA1 inhibitors synergize with BCL-2 blockade.
**Diffuse Large B-Cell Lymphoma**	OPA1 targeted indirectly via OMA1 activation.	OPA1 cleavage contributes to mitochondrial fragmentation and ISR activation.	BTM compounds that activate OMA1 show potent anti-lymphoma activity.

## Data Availability

The original contributions presented in this study are included in the article. Further inquiries can be directed to the corresponding authors.

## Abbreviations

The following abbreviations are used in this manuscript:

AML	Acute myeloid leukemia
ATF4	Activating Transcription Factor 4
ATP	Adenosine Triphosphate
CCA	Cholangiocarcinoma
CLPB	Caseinolytic peptidase B
Cryo-EM	Cryo-electron microscopy
DLBCL	Diffuse large B-cell lymphoma
DOA	Dominant optic atrophy
DRP1	Dynamin-Related Protein 1
EGFR	Epidermal Growth Factor Receptor
EIF2AK1	Eukaryotic translation initiation factor 2-alpha kinase 1
EIF2AK3	eukaryotic translation initiation factor 2-alpha kinase 3
eIF2α	α-subunit of eukaryotic initiation factor 2
ER	Endoplasmic reticulum
ETC	Electron transport chain
FIS1	Mitochondrial Fission 1
GDP	Guanosine Diphosphate
GTP	Guanosine Triphosphate
HCC	Hepatocellular carcinoma
HRI	Heme-Regulated Inhibitor
IMM	Inner mitochondrial membrane
LSCs	Leukemic stem cells
L-OPA1	Long-OPA1
Mfn	Mitofusins
MDs	Molecular Dynamics simulations
MFF	Mitochondrial Fission Factor
MiD	Mitochondrial Dynamics protein
mtDNA	mitochondrial DNA
MTS	Mitochondrial targeting sequence
NSCLC	Non-small cell lung cancer
OMM	Outer mitochondrial membrane
OXPHOS	Oxidative phosphorylation
PDB	Protein Data Bank
ROS	Reactive oxygen species
SAR	Structure-activity relationship
SBVS	Structure-Based Virtual Screening
S-OPA1	Short-OPA1
TM	Transmembrane
TNBC	Triple-negative breast cancer
T-ALL	T-cell acute lymphoblastic leukemia
VDT	Viriditoxin
